# The Role of Phytonutrients in Skin Health

**DOI:** 10.3390/nu2080903

**Published:** 2010-08-24

**Authors:** Julie A. Evans, Elizabeth J. Johnson

**Affiliations:** Jean Mayer US Department of Agriculture Human Nutrition Research Center on Aging at Tufts University, Boston, MA 02111, USA; Email: julie.evans@tufts.edu

**Keywords:** photodamage, vitamin E, flavonoids, carotenoids

## Abstract

Photodamage is known to occur in skin with exposure to sunlight, specifically ultraviolet (UV) radiation. Such damage includes inflammation, oxidative stress, breakdown of the extracellular matrix, and development of cancer in the skin. Sun exposure is considered to be one of the most important risk factors for both nonmelanoma and melanoma skin cancers. Many phytonutrients have shown promise as photoprotectants in clinical, animal and cell culture studies. In part, the actions of these phytonutrients are thought to be through their actions as antioxidants. In regard to skin health, phytonutrients of interest include vitamin E, certain flavonoids, and the carotenoids, β-carotene, lycopene and lutein.

## 1. Introduction

Skin is the largest organ in the human body. It is exposed to environmental insult of which UV light is thought to be the most harmful [1]. UV exposure can cause oxidative stress, inflammation, erythema, breakdown of the extracellular matrix, wrinkling and skin cancer [2,3,4]. In fact, cumulative sun exposure is one of the most important risk factors for both nonmelanoma and melanoma skin cancers [5,6]. 

Over 1 million skin cancers in the US are attributed to UV exposure per year, making it a significant public health concern [7]. The Healthy People 2010 goals include reducing sun exposure as part of the skin cancer reduction goals [8]. Skin cancer can be categorized as nonmelanoma and melanoma. Nonmelanoma cancer includes basal cell carcinoma (BCC) and squamous cell carcinoma (SCC). Nonmelanoma skin cancer has an estimated incidence of over 600,000 cases per year in the US. Approximately 500,000 are BCCs and 100,000–150,000 are SCCs [9]. Melanoma has an estimated incidence of between 11 and 14 per 100,000 cases per year in the US among white populations [10].

There are many ways to protect oneself from UV exposure including avoidance of the sun, wearing protective clothing (hats, long-sleeved shirts, treated fabrics, *etc.*), and topical sunscreen. However, the potential for dietary UV protection is a relatively recent concept. Compared to topical sunscreens which require reapplication and have localized effects, dietary methods are thought to provide continual whole body protection. This promise has led to an explosion in nutrient containing products which are marketed for skin health improvement. Common ingredients of these products, both cosmetic and consumable, are antioxidant nutrients. While a variety of nutrients have been implicated in skin health [11,12], the purpose this review is to evaluate the evidence to date on the role of certain phytonutrients in skin health. Given the implication of oxidative stress being causative in photodamage, antioxidant nutrients are of interest in the prevention of such damage, the nutrients of interest in the study of photoprotection in skin include α-tocopherol, and certain flavonoids and β-carotene, lycopene, and lutein.

## 2. UV Damage

UV light is grouped as UVA (315–400 nm), UVB (280–315 nm) and UVC (220–280 nm), from lowest to highest energy. The Earth’s atmosphere filters out UVC light and of the remaining UV light to which we are exposed, 95% is UVA. The primary focus of UV research has been UVB light, but this is shifting toward UVA light. UVA is of higher wavelength which enables it to penetrate deeper into the skin [13]. UVB is mostly absorbed by the epidermal layer, but UVA affects the dermal layer as well. UVA has demonstrated carcinogenicity in animal models [14], emphasizing the need to understand and prevent the processes by which UVA exerts its biological effects. Clinical changes seen with photoaging, such as roughness, fine wrinkles, spotty hyperpigmentation, vasodilation, and loss of elasticity, are attributed primarily to UVA as well [15]. 

UVA is thought to damage the skin primarily via generating reactive chemical species (ROS) upon absorption of photons by riboflavin, porphyrins and heme-containing proteins [14]. ROS, reactive nitrogen species (RNS) as well as further enzymatic production of ROS have been implicated. Reactive species can damage major biomolecules including DNA, proteins and lipids in various skin strata leading to cytotoxicity, mutations and alterations in cell signaling pathways. In cultured human fibroblasts from foreskin biopsies, UVA induced activity in p38 and c-Jun-N-terminal kinase (JNK) of the mitogen-activated protein kinase (MAPK) family, but not extracellular signal-regulated kinases (ERK)[16]. This activation was attributed to singlet oxygen (^1^O_2_) because of subsequent inhibition by ^1^O_2 _quenchers. This activation can lead to downstream activation of transcription factors that activate stress-inducible genes, specifically matrix metalloproteinase-1 (MMP-1). MMPs are responsible for the degradation of the extracellular matrix in skin. 

## 3. α-Tocopherol

**Figure 1:**
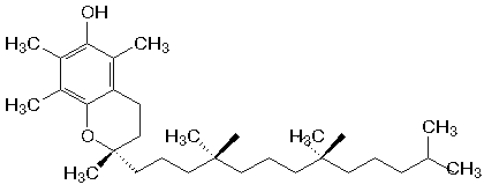
Chemical structure of α-tocopherol (AT).

Vitamin E refers to a family of eight nutrients of which α-tocopherol is the most abundant and biologically active form in the human body (Figure 1). The α form has 3 methyl groups on its chromanol ring and is recognized by the tocopherol transfer protein (TTP) which is responsible for its intracellular transport. This essential lipophilic nutrient is well known for its role as a chain-breaking antioxidant during lipid peroxidation and it protects polyunsaturated fatty acids in cell membranes from oxidation. Vitamin E also has the ability to affect signal transduction and gene expression [17]. Food sources include vegetables, vegetable oils, cereals and nuts [18]. Almonds are a particularly good source of α-tocopherol, containing approximately 26 mg per 100 g [19]. 

α-Tocopherol concentrations in skin can be increased with oral or topical delivery [18] and are decreased with UV exposure [20]. Distribution among the layers of human skin has been determined (Table 1). Within human upper arm stratum corneum, a gradient of highest to lowest α-tocopherol concentration was found from lower to upper layers [21]. α-Tocopherol’s activity as an antioxidant, combined with its presence in human skin, provide biologic plausibility for its role as a photoprotectant in skin. 

**Table 1:** Distribution of α-Tocopherol in human skin compartments.

Skin Layer	Concentration	Reference
Epidermis	31 ± 3.8 nmol /g tissue	Shindo *et al.* [20]
Dermis	16.2 ± 1.1 nmol/g tissue	Shindo *et al.* [20]
Stratum corneum	33 ± 4 nmol/g tissue	Thiele *et al.* [19]
Sebum	76.5 ± 1.5 nmol/g sebum	Thiele *et al.* [21]

### 3.1. Epidemiologic Studies

In a review of epidemiologic literature, McNaughton *et al.* found evidence of a weak protective effect of vitamin E in nonmelanoma carcinoma (BCC and SCC)[22]. Of the 12 studies examined, two identified an inverse relationship between intake and BCC risk while one demonstrated a positive relationship. Six found no relationship between plasma or intake of vitamin E and BCC risk. Four studies found no association between vitamin E and SCC. More recently, two studies in an Australian population reported no association between serum α-tocopherol and nonmelanoma carcinoma but a positive association between dietary supplements, and BCC [23,24]. However, in these two studies a single baseline serum measurement or dietary assessment was compared to cancer incidence during an eight year period. These unexpected findings may reflect the possible inability of a single measure of vitamin E to accurately reflect vitamin E status and a change in eating habit cannot be ruled out over the 8 year period. Furthermore, these studies analyzed the association between cancer, which is a long-term outcome, and short-term markers of vitamin E intake or status such as dietary assessment and serum concentration. Cancer may be closer related to skin concentrations which were not compared in any of these studies.

There are no epidemiological studies assessing the relationship between topically applied α-tocopherol and skin cancer.

### 3.2. Clinical Trials

Clinical trials suggest that dietary vitamin E may act as a photoprotectant with the aid of other antioxidants and topical vitamin E may provide protection as well, although reports have not been entirely consistent.

One study showed subjects treated with 400 IU/d α-tocopherol for eight weeks had reduced skin malondialdehyde (MDA) after UV exposure but this intervention provided no protection from erythema, the reddening of the skin commonly referred to as sunburn [14]. Wernignaus *et al.* [25] showed no photoprotective effect after 6 months of daily dietary supplementation of α-tocopherol acetate (400 IU) in 12 subjects of skin types II-IV. Both clinical measure of minimal erythema dose (MED), the minimum dose of UV necessary to induce erythema in a subject, and histologic measure of sunburn cells were compared. No difference was seen in skin α-tocopherol concentrations between treatment and placebo groups. Plasma α-tocopherol concentrations increased by 65% in the treatment group and 18% in the placebo group, so there was no question of bioavailability. Although a very modest increase in MED was observed in the treatment group after 6 months, the sample size of this study may have been too small to observe significant changes. It is possible that α-tocopherol will provide the most benefit in subjects with a limited range in skin types of increased risk for photodamage (very light to light). Additionally, repetitive UV doses lower than necessary to produce erythema are enough to cause local immunosuppression and skin carcinogenesis [15], suggesting that other markers of damage may be important such as DNA damage.

Other dietary studies have shown vitamin E as a photoprotectant when combined with other antioxidants. Fuchs *et al.* [26] reported no protection when vitamin C (3 g) and α-tocopherol (2 g or 3000 IU) were supplemented individually, but in combination these nutrients provided protection as assessed by increased MED after 50 days of daily supplementation. Eberlein-Konig *et al.* [27] also showed increased MED in subjects supplemented daily with a combination of vitamin C (2 g) and α-tocopherol (1000 IU) after only 8 days. A similar study showed increased MED in subjects supplemented daily with α-tocopherol (1200 IU) for 7 days, but when combined with vitamin C (2 g) a greater increase in MED was observed [28]. Placzek *et al.* [29] demonstrated a photoprotective effect after 3 months of daily dietary supplementation with vitamin C (1 g) and α-tocopherol (500 IU). Participants showed increased resistance to UVB-induced sunburn, as assessed by MED, and protection from DNA damage. This study provides evidence of long-term photoprotection of lower doses than had been previously tested. 

Clinical trials suggest that vitamin E applied topically also provides photoprotection. Chung *et al.* demonstrated topical application of 5% vitamin E (24 hours prior to UV exposure) inhibited UV-induced MMP-12 mRNA by 47% [30]. Other groups have reached opposing conclusions on the protection that vitamin E can provide from erythema. One group demonstrated reduced erythema when vitamin E was applied prior to UV exposure but not after [31]. Another group found vitamin E application after UV exposure protective, but not pretreatment [32]. 

### 3.3. Animal Studies

UVB irradiated mice fed α-tocopheryl acetate had less cancer incidence in a dose-dependent manner [33]. Dietary α-tocopherol esters are readily cleaved and free α-tocopherol is absorbed into the circulatory system. It should be noted that the doses used in this study were considered toxic given that more treated mice died compared to control mice. A more recent experiment tested an oral combination of vitamin C, vitamin E, pycnogenol (a pine bark extract rich in polyphenols and procyanidins), and evening primrose oil in mice [34]. The nutrient cocktail treatment significantly inhibited UVB-induced wrinkling and many associated events, including expressions of MMPs, MAPK and activation of activator protein (AP)-1 transcriptional factor. While these results support a role for nutrient effects in photoprotection, specific effects of vitamin E could not be determined. 

Studies in mice treated topically with α-tocopherol provide strong evidence of photoprotection [18]. Mice treated topically with 25 mg α-tocopherol three times per week for three weeks before and during twelve weeks of UVB irradiation had significantly less incidence of skin cancer compared to untreated mice [35]. Four additional murine studies demonstrated photoprotection, assessed as skin tumor incidence, from topical α-tocopherol treatment [36,37,38,39]. A follow-up study using this same α-tocopherol treatment reduced induction of DNA damage by UVB [40]. Later studies by this group found acetate and succinate esterified forms of α-tocopherol applied in the same manner did not provide the same chemoprevention [41]. This difference may be due to the limited capacity to cleave acetate or succinate when applied topically. This limitation may compromise the antioxidant activity given that ester forms do not have a free hydroxyl group on the aromatic ring necessary for scavenging free radicals. 

An additional 15 animal studies have consistently found topical pretreatment with vitamin E photoprotective using variety of outcome measures including erythema, lipid peroxidation, wrinkling and sunburn cell formation [42,43,44,45,46,47,48,49,50,51,52,53,54,55,56]. 

### 3.4. *In* *Vitro*

A human skin model has previously shown absorption of topical α-tocopherol -glucoside, α-tocopherol and α-tocopherol acetate into epidermal and dermal compartments [57]. α-Tocopherol was not included in this study. Pig skin biopsies have been cultured showing pretreatment with topical α-tocopherol reduced UVB-induced cytotoxicity, apoptosis and lipid peroxidation [20]. In cultured mouse skin, media pretreatment of 9 μM α-tocopherol-6-*O*-phosphate, a water-soluble form of α-tocopherol, reduced UVB-induced apoptosis and lipid peroxidation [58]. Media pretreatment of α-tocopherol in human melanocytes prevented UVA- and UVB-induced glutathione loss and diminished apoptosis [59]. 

## 4. Flavonoids

**Figure 2:**
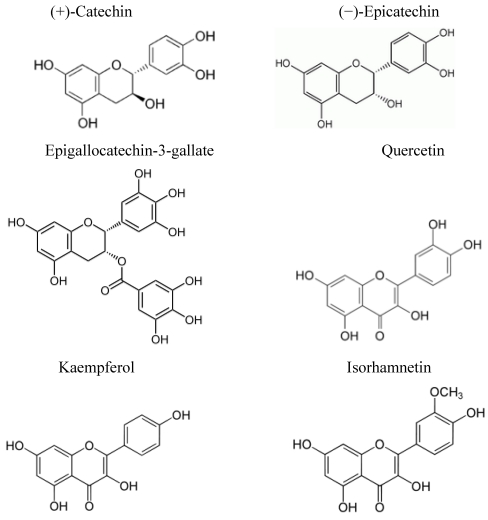
Chemical structure of predominant polyphenols.

Flavonoids are a family of over 5000 compounds found in plants. Flavonoid structure is based on a flavane nucleus, which contains two benzene rings separated by an oxygen containing pyrane ring. Individual flavonoids compounds have been reported to be radical scavengers, UVA absorbent, cytoprotective, anti-inflammatory anti-apoptotic, and to inhibit DNA damage and to affect cellular signaling pathways [60,61,62]. Collectively, flavonoids act *in vitro* as antioxidants and induce quinone reductase activity, a marker of chemopreventive activity [63]. Addition of almonds, a rich source of flavonoids, to human diet has reduced biomarkers of oxidative stress [64]. Predominant dietary flavonoids include isorhamnetin, catechin, kaempferol, epicatechin, quercetin and epigallocatechin-3-gallate (EGCG)(Figure 2)[65].

Absorption of flavonoids to human skin has not been tested. However, radioactivity was detected in the skin of mice fed ^3^H-labeled EGCG, a closely related flavanol found in green tea [66]. From a single topical dose of EGCG, maximal concentrations of 1,366 and 411 ng/mL were measured in mouse epidermis and dermis, respectively [67]. 

### 4.1. Epidemiologic Studies

Epidemiologic evidence suggests an association between flavonoid-rich foods and skin cancer incidence. A case-control study in an Italian population found a negative correlation between cutaneous melanoma and daily tea drinking, high consumption of vegetables, particularly carrots, cruciferous and leafy vegetables, and fruits, especially citrus [68]. In the “Food Habits in Later Life” study, tea consumption was negatively correlated with actinic skin damage in Anglo-Celtic Australians [69]. 

### 4.2. Clinical Trials

The acute and long-term photoprotective effects of consumption of a flavanol-rich cocoa beverage was studied in double-blind, placebo controlled clinical trials. Epicatechin and its stereoisomer catechin are the major flavanols found in this beverage, contributing 61 and 20 mg/serving, respectively to a total of 329 mg cocoa flavanols. Two hours after a single dose of the flavanol-rich cocoa, dermal blood flow and oxygen saturation of hemoglobin significantly increased by 1.7- and 1.8-fold, respectively [70]. Long-term, daily ingestion of the same flavanol-rich cocoa beverage decreased UV-induced erythema by 15% after only six weeks [71]. After twelve weeks, additional protection was evident as erythema was decreased by 25%. Blood flow of cutaneous and subcutaneous tissues, skin density and skin hydration were also increased while skin roughness and scaling were significantly decreased. None of these skin improvements was observed in the control group. 

Green tea contains the flavanols catechin and epicatechin. An eight week clinical trial evaluating the clinical and histological effects of combined topical and oral green tea supplementation (300 mg twice daily) found improvements in elastic tissue content [72]. However no significant differences in clinical grading of appearance were found. All subjects were asked to apply sunscreen daily which may have attenuated differences between groups. 

Immunosuppression was inhibited in participants after a single topical application of either green or white tea treatment (2.5 mg/cm^2^) as evaluated by hypersensitivity to dinitrochlorobenzene [73]. The same topical treatments prevented DNA damage. Another study found a single topical dose of green tea treatment (1–4 mg/2.5 cm^2^) protective of DNA damage, in a dose-dependent manner [74]. Long-term topical application of green tea treatment significantly reduced UV-induced p53 expression and apoptosis in keratinocytes, but not erythema [75].

Topical application of an extract of *Culcitium reflexum* H.B.K. leaves which contain isorhamnetin, quercetin and kaempferol significantly decreased erythema when applied after UVB irradiation [76]. 

### 4.3. Animal Studies

Investigations of foods rich in flavanoid nutrients demonstrate their efficacy as photoprotectants against UV carcinogenesis in animal models. Grape seed proanthocyanidins are polymers of flavanol monomers such as catechin and epicatechin [74]. In SKH-1 hairless mice, dietary supplementation with these compounds resulted in a dose-dependent reduction in photocarcinogenesis. Green tea as well as black tea consumption also resulted in significantly fewer UV-induced tumors in mice [77]. The administration of green tea polyphenols in drinking water or the topical application of EGCG, a major tea flavanol, also induced partial regression or inhibition of tumor growth of established skin papillomas in mice [78]. Gensler *et al.* also investigated EGCG in BALB/cAnNHsd mice. They found induction of skin tumors by UV irradiation was significantly reduced by topical but not by oral treatment of EGCG [79]. Other research has focused on acute photoprotection of EGCG. In Wistar rats, topical EGCG treatment for 30 minutes prior to UVA irradiation significantly decreased sunburn cell occurrence and dermo-epidermal activation compared to untreated rats [80]. The same protection was not observed with topical EGCG application 30 minutes after UVA exposure. UVA-induced oxidative stress was significantly attenuated in Sprague-Dawley rats by intraperitoneal injections of quercetin prior to UV exposure [81]. Markers of oxidative stress included increased plasma malondialdehyde (a product of lipid peroxidation), decreased erythrocyte glutathione peroxidase and reductase activities, and decreased catalase and superoxide sidmutase activities. Topical quercetin treatment was found protective of UVB-induced oxidative stress in mouse skin as well [82]. 

Positive photoprotective results from the above animal studies lead researchers to investigate the mechanism of protection. Dietary supplementation of green tea polyphenols in mice, during 8 weeks of UVB irradiation, inhibited UV-induced expression of MMPs, including MMP-2, -3, -7, and -9 [83]. All of these MMPs have been shown to be involved in the degradation of types-I and -III collagen fragments generated by collagenases and type IV collagen of the basement membrane. 

### 4.4. *In Vitro* Studies

In human dermal fibroblasts, both quercetin and kaempferol inhibited MMP-1 (collagenase) activity and expression induced by UVA [34]. EGCG inhibited MMP-2 and -9 activity (gelatinases)[84]. Adult human skin fibroblasts and normal human epidermal keratinocytes, cultured separately, had significantly less DNA damage from UVA irradiation when treated with EGCG [85]. 

*Ex vivo* cultured human skin experiments demonstrated pretreatment with topical green and white tea (2 mg/cm^2^) provided protection from UV induced immunosuppression, measured by depletion of Langerhans cells [73]. Another *ex vivo* human skin experiment found topical cocoa polyphenol extract (containing catechin and epicatechin) improved skin structure after five days of application [86]. Glycosaminoglycans increased as well as collagen I, II and IV expression, indicating improved elasticity and skin tonus, respectively. In UVA exposed artificial skin models, composed of differentiated epidermis on a dermal substitute, pretreatment with topical EGCG decreased expression of MMP-1 and -3 and activity of MMP-2 and -9 [87]. Tissue inhibitor of metalloproteinase-1 (TIMP-1) expression increased simultaneously. 

## 5. Carotenoids

Carotenoids are a family of compounds of over 600 fat-soluble plant pigments [88]. Fruits and vegetables are major sources of carotenoids [89]. Carotenoids are considered to be efficient scavenger of singlet molecular oxygen [90,91]. In fact, β-carotene (a major dietary carotenoid), at physiological concentrations, has been demonstrated to interact with UVA effects in keratinocytes by mechanisms that included singlet molecular oxygen quenching [92]. Other dietary major dietary carotenoids include lycopene, and lutein [89]. 

Carotenoids concentrations in human skin have been reported to range from 0.2 to 0.6 nmol/g wet weight [93]. Carotenoid skin concentrations have been shown to vary by location on the body (forehead > palm > dorsal > inside arm = back of hand)[94,95,96,97]. Further, skin concentrations can be increased with supplementation [71] and are lower in persons with oxidative stress (e.g., smokers)[94]. Similarly, plasma and skin carotenoids decrease with UV exposure [98,99]. Based on the presence of carotenoids in human skin and the known biologic activities of carotenoids, including quenching singlet oxygen species, carotenoids have been hypothesized to protect the skin from UV damage. 

### 5.1. Epidemiological Studies and Fruit &amp; Vegetable Intakes

Epidemiological studies have provided some evidence that fruit and vegetable intake may improve skin health and decrease the risk of skin cancer. However, in most cases, the specific effect of an individual carotenoid was not evaluated. Nonetheless, they provide evidence to support a role for carotenoids, and other phytonutrients, in skin health.

In a cross-sectional study investigating the association between skin wrinkling and dietary intake, a significant negative association was found with eggs and green leafy vegetables [69]. Eggs are a highly bioavailable source or lutein [100] and green leafy vegetables are good sources of both β-carotene and lutein [89]. This relationship between carotenoids intake and skin wrinkling may be due to an ability to prevent extracellular matrix breakdown [101]. An association of green leafy vegetables with decreased risk of skin cancer has also been reported [102]. In this prospective study, a higher consumption of fruits and vegetables was related to decreased risk of SCC risk by 54%. This protective effect was mostly explained by the association with green leafy vegetables. 

In an observational study of an Italian population, high intake (at least 3 times per week) of dark green leafy vegetables was associated with decreased risk of cutaneous melanoma after adjustment for sex, age, education, hair color, skin phototypes, number of nevi, presence of freckles and sunburns in childhood [68]. In another observational study which looked at BCC and SCC, a decreased risk of SCC in persons with a history of skin cancer and high dietary intake of lutein and zeaxanthin was reported [23]. Other studies show a mix of results including no association between serum lutein/zeaxanthin and BCC or SCC [24] and a positive association between serum lutein/zeaxanthin and risk of SCC in participants with a history of BCC [103]. Dietary lutein (combined with zeaxanthin) showed a slightly positive relationship with SCC and BCC incidence in women [104,105]. However, vitamins A, C, and E, folate and total carotenoids were also found to have a positive relationship. The mixed results of these studies could be due to limitations in dietary assessment or individual variability in the ability to accumulate certain phytonutrients in skin tissue.

### 5.2. β-Carotene

**Figure 3:**
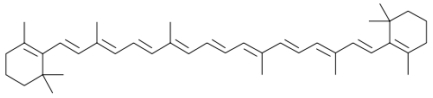
Chemical structure of β-carotene.

β-Carotene is in the carotene class of carotenoids (Figure 3). It is a strongly-colored red-orange pigment. As a carotene with beta-rings at both ends, it is the most common form of carotene. It is a precursor of vitamin A. Rich sources of β-carotene include yellow and orange fruits, such as mangoes and papayas, orange root vegetables such as carrots and yams and in green leafy vegetables such as spinach, kale, sweet potato leaves, and sweet gourd leaves [89]. β-Carotene inhibits free radical and singlet oxygen-induced lipid peroxidation in liposomes and the biologic system. It is a photoprotective agent and is thought to quench photochemical reactions in the epidermis involving singlet oxygen and oxygen radicals generated by UV exposure [106]. 

#### 5.2.1. Epidemiologic Studies

The epidemiologic studies that have evaluated a relationship between β-carotene and skin cancer risk have provided little support for a protective role of this carotenoid. No studies have found a relationship between plasma β-carotene and risk of BCC [107,108,109,110,111,112,113,114]. The few studies have examined SCC also found no relationships [110,112,114,115]. However, in a prospective study among 1001 randomly selected adults, dietary intake of β-carotene in the second tertile was associated with increased risk of BCC, with no trend [116].

#### 5.2.2. Clinical Trials

The photoprotectiveness of β-carotene was first demonstrated with a study of healthy volunteers receiving 180 mg β-carotene/day for 10 weeks or a placebo [117]. Compared to the placebo group, threshold MED was significantly higher in the supplemented group, although there was no significant difference between the two groups in the degree of erythema. Similarly, in a placebo-controlled study involving 30 mg/day β-carotene for 10 weeks, there was a significantly decrease in the intensity of erythema induced by sunlight [118]. Lee *et al.* examined the effects of increasing does of β-carotene (30–90 mg/day for 24 weeks) on UV-induced erythema [119]. In a study evaluating the immune response to supplementation with either 30 mg/day β-carotene or a placebo was studied in 24 men (19–30 years)[120]. Subjects ingested the supplement with a low carotenoid diet for 70 days. From day 28, the subjects were exposed to UV light 12 times over 16 days. No reductions in plasma carotenoid concentrations were observed in either group. Delayed-type hypersensitivity (DTH) skin responses decreased in the placebo group but were unchanged in the supplemented group. The investigators concluded that β-carotene protected against photosuppression of the immune system and that BC supplementation may be beneficial in those with compromised immune function.

Herraiz *et al.* conducted a similar study involving 32 elderly men (55–79 years) receiving either 30 mg/day β-carotene or a placebo with a low carotenoid diet for 47 days [121]. Repeated exposure to UV light resulted in a decrease in DTH skin responses in the placebo group but not in the supplemented group and plasma β-carotene concentrations were unchanged. Higher plasma β-carotene concentrations were related to higher resistance to immunosuppression after exposure to UV light.

The photoprotectiveness of β-carotene(24 mg/day) was compared to that of a carotenoid mixture of β-carotene, lycopene and lutein (8 mg/day each) in a 12 week intervention trial [122]. Erythema intensity after irradiation with a solar light simulator was determined at baseline and at 6 and 12 weeks of supplementation. The intensity of erythema 24 hours after irradiation was significantly decreased to a similar level in both groups. 

A recent meta-analysis of controlled clinical trials reported an overall protective effect of β-carotene supplements against sunburn [123]. Although the studies to date suggest that high dose supplementation with β-carotene may be of benefit to skin health, there is a question of safety with these high intakes. Two intervention trials involving individuals at high risk for cancer, there was a significantly increased incidence of lung cancer in those that received the β-carotene supplement [124,125].

#### 5.2.3. Animal Studies

Minami *et al.* evaluated the role of lipid peroxidation products in MMP-9 activation and the effect of dietary β-carotene in MMP-9 activation. In this study, hairless mice were subjected to periodic UVA-irradiation for 8 weeks [126]. The amount of peroxidized cholesterol (primary product of lipid peroxidation in biomembranes) in the skin significantly increased by the UV exposure. The activity and protein level of MMP-9 were elevated, as well as the wrinkling and sagging formation. Adding β-carotene to the diet of the mice during the period of irradiation suppressed the activity and expression of MMP-9 along with the wrinkling and sagging formation. The amount of peroxidized cholesterol was also significantly lowered. These investigators concluded that dietary β-carotene prevents the expression of MMP-9, in part by inhibiting photodynamic action involved in the formation of peroxidized cholesterol, the result of which was less wrinkling and sagging.

#### 5.2.4. *In Vitro* Studies

β-Carotene, along with the carotenoids astaxanthin, and canthaxanthin, were evaluated for systemic photoprotection in human dermal fibroblasts [127]. Fibroblasts were exposed to moderate doses of UVA which resulted in apoptosis and increased oxidative damage and expression of oxidative stress-response enzymes. When the carotenoids were delivered to fibroblasts 24 hours before exposure to exposure to UVA, astaxanthin exhibited a pronounced photoprotective effect and counteracted all of the measured UVA-induced alterations to a significant extent. β-Carotene only partially prevented the UVA-induced decline of catalase and superoxide dismutase activities, but it increased membrane damage and stimulated heme-ozygenase-1 expression. Furthermore, β-carotene dose-dependently induced caspase-3 activity following UVA exposure. However, canthaxathin had no effect on oxidative damage, except for heme-oxygenase-1 expression, which was increased. The data suggest that among the carotenoids tested, astaxanthin is the most protective towards photo-oxidative changes in cell culture.

### 5.3. Lycopene

**Figure 4:**
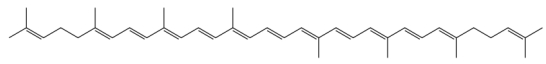
Chemical structure of lycopene.

Lycopene (Figure 4) is a carotene found in primarily in tomatoes but also in other red fruits and vegetables, such as red carrots, watermelons and papayas [89]. Unlike β-carotene, lycopene has no vitamin A activity [128]. Lycopene is considered to be the most the most efficient biological carotenoid singlet oxygen quencher [129]. A suggestion that lycopene may be protective in skin health comes from the work of Darvin *et al.* in which it was reported that there was a significant correlation between skin roughness and lycopene concentrations in the skin [130]. Topical application of lycopene contained in monoglyceride or triglyceride microemulsions was found to result in lycopene penetration in the stratum corneum (6- and 3.6-fold, respectively) and in viable layers of porcine ear skin (from undetected to 172.6 and 103.1 ng/cm^2^, respectively) compared to a control solution. Furthermore, the antioxidant activity of skin treated with lycopene was found to be 10-fold higher than untreated skin [131]. 

#### 5.3.1. Epidemiologic Studies

Serum or plasma concentrations of lycopene were found to not be related with either BCC or SCC [110,113,114]. These results are contrary to observations in clinical trials (see below). 

#### 5.3.2. Clinical Trials

Intervention trials have been conducted to evaluate the effect of lycopene supplementation on erythema prevention [132,133]. The lycopene intervention was for 10–12 weeks and volunteers consumed one of five sources of lycopene: tomato paste (16 mg/day lycopene); carrot juice (10 mg/day lycopene, 5.1 mg/day β-carotene; lycopene supplement (9.8 mg/day lycopene, 0.4 mg/day β-carotene); tomato extract (8.2 mg/day lycopene, 0.4 mg β-carotene); or synthetic lycopene (10.2 mg/day lycopene). In all studies serum lycopene significantly increased at study end. Skin carotenoids levels also increased with all interventions. However, only total carotenoids could be quantitated, as reflection spectroscopy was the method of assessment. With the exception of synthetic lycopene, all lycopene treatments were found to be effective in being photoprotective, as measured by erythema formation. Of note, is that that the synthetic lycopene was the only intervention to contain no other carotenoids. Additionally, the tomato-based interventions contain other constituents including the carotenoids phytofluene and phytoene. It is possible that these other carotenoid contribute to photoprotectiveness since they absorb light in the UV range. 

Darvin *et al.* evaluated the relationship between skin lycopene concentrations and skin roughness [130]. Lycopene was analyzed quantitatively on the forehead skin of 20 volunteers aged between 40 and 50 years. There was, no significant correlation between age and skin roughness but, a significant correlation was obtained between the skin roughness and the lycopene concentration (R = 0.843).

These findings suggest that higher levels of lycopene in the skin effectively lead to lower levels of skin roughness, however, since other antioxidants were not evaluated, it if difficult to conclude that this relationship was specific to lycopene. 

#### 5.3.3. *In Vitro* Studies

Lycopene has been shown to inhibit proliferation of several types of cancer cells through arrest of tumor cell-cycle progression, IGF-1 (insulin-like growth factor 1) signaling transduction, and induction of apoptosis [134,135]. Wu *et al.* [136] found that lycopene inhibited PDGF-BB (platelet-derived growth factor-BB)-induced signaling and cell migration in human cultured skin fibroblasts through direct binding to PDGF-BB. Trapping of PDGF by lycopene also compromised melanoma-induced fibroblast migration and attenuated signaling transduction in fibroblasts simulated by melanoma-derived conditioned medium. These findings suggest that lycopene may interfere with tumor–stroma interactions. These investigators concluded that the trapping activity of lycopene on PDGF suggests that it may act as an inhibitor on stromal cells, tumor cells and their interactions, which may contribute to its anti-tumor activity.

### 5.4. Lutein

**Figure 5:**
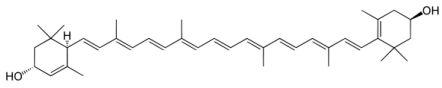
Chemical structure of lutein.

Lutein is a member of the xanthophyll class of carotenoids (Figure 5). Carotenoids are a family of more than 600 lipid-soluble compounds that are plant pigments. All carotenoids have a backbone composed of conjugated double bonds which is essential for their antioxidant activity. Xanthophylls, the most polar of carotenoids, are unique to this family because of their functional hydroxyl groups. 

Lutein is commonly found in fruits and vegetables. The richest source of lutein is leafy green vegetables but also can be found in significant amounts in broccoli, corn and peas [137,138]. Although levels are not high, lutein is also found in egg yolk, which is considered to be a highly bioavailable source of lutein [139]. There is no established Dietary Recommended Intake (DRI) for lutein, but 6 mg is associated with a reduced risk of cataracts and age-related macular degeneration [140,141,142]. Most Americans only get 1 to 2 mg/day [143,144,145,146].

Lutein has a well-established role in the eye health, acting as an antioxidant and absorbing blue light. Lutein also has an established presence in epidermal and dermal compartments of the skin and contributes to skin color [97]. 

#### 5.4.1. Epidemiologic Studies

Epidemiological studies have provided some evidence that the level of lutein intake may improve skin health and decrease the risk of skin cancer. 

In a cross-sectional study investigating the association between skin wrinkling and dietary intake, a significant negative association was found with eggs and green leafy vegetables [69]. This relationship between lutein intake and skin wrinkling may be due to lutein’s ability to prevent extracellular matrix breakdown [101]. An association of green leafy vegetables with decreased risk of skin cancer has also been reported [102]. In this prospective study, a higher consumption of fruits and vegetables was related to decreased risk of SCC risk by 54%, but this protective effect was mostly explained by the association with green leafy vegetables. 

In an observational study of an Italian population, high intake (at least 3 times per week) of lutein-rich dark green leafy vegetables was associated with decreased risk of cutaneous melanoma after adjustment for sex, age, education, hair color, skin phototypes, number of nevi, presence of freckles and sunburns in childhood [68]. In another observational study which looked at BCC and SCC, a decreased risk of SCC in persons with a history of skin cancer and high dietary intake of lutein and zeaxanthin was reported [23]. Other studies show a mix of results including no association between dietary lutein-zeaxanthin and BCC or SCC [24] and a positive association between serum lutein-zeaxanthin and risk of SCC in participants with a history of BCC [103]. Dietary lutein (combined with zeaxanthin) showed a slightly positive relationship with SCC and BCC incidence in women [104,105]. However, vitamins A, C, and E, folate and total carotenoids were also found to have a positive relationship. The mixed results of these studies could be due limitations in assessment of dietary lutein or individual variability in the ability to accumulate lutein in skin tissue, as has been noted for the macula [147,148,149].

#### 5.4.2. Clinical Trials

Results from clinical trials looking at photoprotection support a role for lutein in skin health. One of the earliest of these studies showed oral supplementation with 24 mg carotenoids/day (including 8 mg/day each β-carotene, lutein and lycopene) improved UV-induced erythema in humans after only 6 weeks of supplementation in a 12 week trial [71]. Others have shown an increase in skin hydration and superficial skin lipids with twice daily oral supplementation (45 mg vitamin C, 5 mg tocopherol, 3 mg lutein and 2.5 mg α-lipoic acid). Also, skin lipid peroxides (a measure of oxidation) were decreased [150]. While these studies suggest a role for antioxidants in skin health, it is difficult to determine the individual contribution of lutein.

Palombo *et al.* [150] provide strong evidence that increased lutein intake may improve skin health. This double-blind placebo controlled trial was designed to study the efficacy of oral supplementation (lutein 10 mg/day, zeaxanthin 0.6 mg/day) or topical application (lutein 100 ppm/day, zeaxanthin 4 ppm/day) on five physiology parameters (surface lipids, hydration, photoprotective activity, skin elasticity, skin lipid peroxidation). This was a 12 week intervention. Oral or topical administration of these carotenoids significantly improved these measures, with oral administration providing better protection when measured by changes in lipid peroxidation and photoprotective activity in the skin following UV irradiation. However, the combined oral and topical administration provided the greatest protection.

#### 5.4.3. Animal Studies

A study in hairless mice has shown protection from photocarcinogenesis (both number and size of tumors) with lutein supplementation [151]. In this study, mice received either a lutein/zeaxanthin-supplemented diet or a standard nonsupplemented diet. Dorsal skin of female Skh-1 hairless mice was exposed to UVB radiation in doses required for photoaging and for photocarcinogenesis. Clinical evaluations were performed weekly, and the animals were sacrificed 24 h after the last UVB exposure. For photoaging experiments, skin fold thickness, suprapapillary plate thickness, mast cell counts and dermal desmosine content were evaluated. For photocarcinogenesis, samples of tumors larger than 2 mm were analyzed for histological characterization, hyperproliferation index, tumor multiplicity, total tumor volume and tumor-free survival time. Results of the photoaging experiment found that skin fold thickness and number of infiltrating mast cells following UVB irradiation were significantly less in lutein/zeaxanthin-treated mice when compared to irradiated animals on the standard diet. In the photocarcinogenesis experiment, tumor-free survival time increased, tumor multiplicity decreased and total tumor volume decreased in lutein/zeaxanthin-treated mice compared to control irradiated animals fed the standard diet. The results from this study suggest that dietary lutein/zeaxanthin supplementation protects the skin against UVB-induced photoaging and photocarcinogenesis.

#### 5.4.4. *In* *Vitro*

Lutein protected rat kidney fibroblasts from UVA-induced oxidative stress, including decreases in the antioxidant enzymes, catalase and superoxide dismutase [152]. Furthermore, in human lens epithelial cells, pretreatment with lutein decreased UVB-induced lipid peroxidation and activation of p38 and JNK [153]. Lutein significantly inhibited MMP-1 expression in dermal fibroblasts and melanoma cells [101]. Results from these *in vitro* studies lend mechanistic support to a lutein’s role in skin health.

## 6. Conclusions and Summary

Skin cancer is a significant public health concern. The skin is vulnerable to UV damage, which can lead to cancer. Possible mechanism by which this occurs include oxidative damage and inflammation. A diet rich in phytonutrients, including α-tocopherol, flavonoids, β-carotene, lycopene and lutein, may produce continual whole body protection from such damage. Epidemiological studies support a role for diets high in these food components and decreased risk of photoaging and cancer. However, the data are not entirely consistent. Stronger evidence comes from intervention studies which find supplementation with α-tocopherol, flavonoids, β-carotene, lycopene, lutein and various nutrient combinations can protect from shorter-term markers of UV damage including lipid peroxidation, erythema, MMP expression, DNA damage, and apoptosis. Similar results are observed from topical treatments of these nutrients. Further investigation of optimal doses and mechanism of protection are needed to better target and prevent photodamage with dietary and/or topical treatments. 

## References

[1] Lippens S., Hoste E., Vandenabeele P., Agostinis P., Declercq W. (2009). Cell death in the skin. Apoptosis.

[2] Boelsma E., Hendriks H.F., Roza L. (2001). Nutritional skin care: health effects of micronutrients and fatty acids. Am. J. Clin. Nutr..

[3] Iddamalgoda A., Le Q.T., Ito K., Tanaka K., Kojima H., Kido H. (2008). Mast cell tryptase and photoaging: possible involvement in the degradation of extra cellular matrix and basement membrane proteins. Arch. Dermatol. Res..

[4] Mudgil A.V., Segal N., Andriani F., Wang Y., Fusenig N.E., Garlick J.A. (2003). Ultraviolet B irradiation induces expansion of intraepithelial tumor cells in a tissue model of early cancer progression. J. Invest. Dermatol..

[5] Gloster H.M., Brodland D.G. (1996). The epidemiology of skin cancer. Dermatol. Surg..

[6] Miller A.J., Mihm M.C. (2006). Melanoma. N. Engl. J. Med..

[7] Greenlee R.T., Hill-Harmon M.B., Murray T., Thun M. (2001). Cancer statistics, 2001. CA Cancer J. Clin..

[8] (2010). Healthy People 2010, Volume I. Office of Disease Prevention and Health Promotion. U.S. Department of Health and Human Services: Rockville, MD, USA,. http://www.healthypeople.gov/Document/HTML/Volume1/03Cancer.htm.

[9] Silverberg E., Boring C.C., Squires T.S. (1990). Cancer statistics, 1990. CA. Cancer J. Clin..

[10] National Cancer Institute. (1993). SEER Program Cancer Facts and Figures.SEER Cancer Statistics Review 1973-1990; NIH publication no. 93-2789.

[11] Boelsma E., Hendricks H.F.J., Roza L. (2001). Nutritional skin care: health effects of micronutrients and fatty acids. Am. J. Clin. Nutr..

[12] Chiu A., Kimball A.B. (2003). Topical vitamins, minerals and botanical ingredients as modulators of environmental and chronological skin damage. Br. J. Dermatol..

[13] Bruls W.A., Slaper H., van der Leun J.C., Berrens L. (1984). Transmission of human epidermis and stratum corneum as a function of thickness in the ultraviolet and visible wavelengths. Photochem. Photobiol..

[14] McMillan D.C., Talwar D., Sattar N., Underwood M., O'Reilly D., McArdle C. (2002). The relationship between reduced vitamin antioxidant concentrations and the systemic inflammatory response in patients with common solid tumours. Clin. Nutr..

[15] Touitou E., Godin B. (2008). Skin nonpenetrating sunscreens for cosmetic and pharmaceutical formulations. Clin. Dermatol..

[16] Klotz L.O., Pellieux C., Briviba K., Pierlot C., Aubry J.M., Sies H. (1999). Mitogen-activated protein kinase (p38-, JNK-, ERK-) activation pattern induced by extracellular and intracellular singlet oxygen and UVA. Eur. J. Biochem..

[17] Zingg J.M. (2007). Modulation of signal transduction by vitamin E. Mol. Aspects Med..

[18] Thiele J.J., Ekanayake-Mudiyanselage S. (2007). Vitamin E in human skin: organ-specific physiology and considerations for its use in dermatology. Mol. Aspects Med..

[19] USDA Agricultural Research Service. (1998). 1994–1996 Continuing survey of food intakes by individuals and diet and health knowledge survey.

[20] Rijnkels J.M., Moison R.M., Podda E., van Henegouwen G.M. (2003). Photoprotection by antioxidants against UVB-radiation-induced damage in pig skin organ culture. Radiat. Res..

[21] Thiele J.J., Traber M.G., Packer L. (1998). Depletion of human stratum corneum vitamin E: an early and sensitive *in vivo* marker of UV induced photo-oxidation. J. Invest. Dermatol..

[22] McNaughton S.A., Marks G.C., Green A.C. (2005). Role of dietary factors in the development of basal cell cancer and squamous cell cancer of the skin. Cancer Epidemiol. Biomarkers Prev..

[23] Heinen M.M., Hughes M.C., Ibiebele T.I., Marks G.C., Green A.C., van der Pols J.C. (2007). Intake of antioxidant nutrients and the risk of skin cancer. Eur. J. Cancer.

[24] van der Pols J.C., Heinen M.M., Hughes M.C., Ibiebele T.I., Marks G.C., Green A.C. (2009). Serum antioxidants and skin cancer risk: an 8-year community-based follow-up study. Cancer Epidemiol. Biomarkers Prev..

[25] Werninghaus K., Meydani M., Bhawan J., Margolis R., Blumberg J.B., Gilchrest B.A. (1994). Evaluation of the photoprotective effect of oral vitamin E supplementation. Arch. Dermatol..

[26] Fuchs J., Kern H. (1998). Modulation of UV-light-induced skin inflammation by D-alpha-tocopherol and L-ascorbic acid: a clinical study using solar simulated radiation. Free Radic. Biol. Med..

[27] Eberlein-Konig B., Placzek M., Przybilla B. (1998). Protective effect against sunburn of combined systemic ascorbic acid (vitamin C) and d-alpha-tocopherol (vitamin E). J. Am. Acad. Dermatol..

[28] Mireles-Rocha H., Galindo I., Huerta M., Trujillo-Hernandez B., Elizalde A., Cortes-Franco R. (2002). UVB photoprotection with antioxidants: effects of oral therapy with d-alpha-tocopherol and ascorbic acid on the minimal erythema dose. Acta Derm. Venereol..

[29] Placzek M., Gaube S., Kerkmann U., Gilbertz K.P., Herzinger T., Haen E., Przybilla B. (2005). Ultraviolet B-induced DNA damage in human epidermis is modified by the antioxidants ascorbic acid and D-alpha-tocopherol. J. Invest. Dermatol..

[30] Chung J.H., Seo J.Y., Lee M.K., Eun H.C., Lee J.H., Kang S., Fisher G.J., Voorhees J.J. (2002). Ultraviolet modulation of human macrophage metalloelastase in human skin *in vivo*. J. Invest. Dermatol..

[31] Dreher F., Maibach H. (2001). Protective effects of topical antioxidants in humans. Curr. Prob. Dermatol..

[32] Montenegro L., Bonina F., Rigano L., Giogilli S., Sirigu S. (1995). Protective effect evaluation of free radical scavengers on UVB induced human cutaneous erythema by skin reflectance spectrophotometry. Int. J. Cosmet. Sci..

[33] Gerrish K.E., Gensler H.L. (1993). Prevention of photocarcinogenesis by dietary vitamin E. Nutr. Cancer.

[34] Chong E.W.T., Wong T.Y., Kreis A.J., Simpson J.A., Guymer R.H. (2007). Dietary antioxidants and primary prevention of age related macular degeneration: systematic review and meta-analysis. BMJ.

[35] Gensler H.L., Magdaleno M. (1991). Topical vitamin E inhibition of immunosuppression and tumorigenesis induced by ultraviolet irradiation. Nutr. Cancer.

[36] Bissett D.L., Chatterjee R., Hannon D.P. (1990). Photoprotective effect of superoxide-scavenging antioxidants against ultraviolet radiation-induced chronic skin damage in the hairless mouse. Photodermatol. Photoimmunol. Photomed..

[37] Bissett D.L., Chatterjee R., Hannon D.P. (1992). Protective effect of a topically applied anti-oxidant plus an anti-inflammatory agent against ultraviolet radiation-induced chronic skin damage in the hairless mouse. J. Soc. Cosmet. Chem..

[38] Bissett D.L., Hillebrand G.G., Hannon D.P. (1989). The hairless mouse as a model of skin photoaging: its use to evaluate photoprotective materials. Photodermatol.

[39] Burke K.E., Clive J., Combs G.F., Commisso J., Keen C.L., Nakamura R.M. (2000). Effects of topical and oral vitamin E on pigmentation and skin cancer induced by ultraviolet irradiation in Skh:2 hairless mice. Nutr. Cancer.

[40] Chen W., Barthelman M., Martinez J., Alberts D., Gensler H.L. (1997). Inhibition of cyclobutane pyrimidine dimer formation in epidermal p53 gene of UV-irradiated mice by alpha-tocopherol. Nutr. Cancer.

[41] Gensler H.L., Aickin M., Peng Y.M., Xu M. (1996). Importance of the form of topical vitamin E for prevention of photocarcinogenesis. Nutr. Cancer.

[42] Beijersbergen van Henegouwen G.M., Junginger H.E., de Vries H. (1995). Hydrolysis of RRR-alpha-tocopheryl acetate (vitamin E acetate) in the skin and its UV protecting activity (an *in vivo* study with the rat). J. Photochem. Photobiol. B.

[43] Stoyanovsky D.A., Goldman R., Darrow R.M., Organisciak D.T., Kagan V.E. (1995). Endogenous ascorbate regenerates vitamin E in the retina directly and in combination with exogenous dihydrolipoic acid. Curr. Eye Res..

[44] Evelson P., Ordonez C.P., Llesuy S., Boveris A. (1997). Oxidative stress and *in vivo* chemiluminescence in mouse skin exposed to UVA radiation. J. Photochem. Photobiol. B.

[45] Jurkiewicz B.A., Bissett D.L., Buettner G.R. (1995). Effect of topically applied tocopherol on ultraviolet radiation-mediated free radical damage in skin. J. Invest. Dermatol..

[46] Khettab N., Amory M.C., Briand G., Bousquet B., Combre A., Forlot P., Barey M. (1988). Photoprotective effect of vitamins A and E on polyamine and oxygenated free radical metabolism in hairless mouse epidermis. Biochimie.

[47] Lin J.Y., Selim M.A., Shea C.R., Grichnik J.M., Omar M.M., Monteiro-Riviere N.A., Pinnell S.R. (2003). UV photoprotection by combination topical antioxidants vitamin C and vitamin E. J. Am. Acad. Dermatol..

[48] Lopez-Torres M., Thiele J.J., Shindo Y., Han D., Packer L. (1998). Topical application of alpha-tocopherol modulates the antioxidant network and diminishes ultraviolet-induced oxidative damage in murine skin. Br. J. Dermatol..

[49] McVean M., Liebler D.C. (1997). Inhibition of UVB induced DNA photodamage in mouse epidermis by topically applied alpha-tocopherol. Carcinogenesis.

[50] McVean M., Liebler D.C. (1999). Prevention of DNA photodamage by vitamin E compounds and sunscreens: roles of ultraviolet absorbance and cellular uptake. Mol. Carcinog..

[51] Potapenko A., Abijev G.A., Pistsov M., Roshchupkin D.I., Vladimirov Yu A., Pliquett F., Ermolayev A.V., Sarycheva I.K., Evstigneeva R.P. (1984). PUVA-induced erythema and changes in mechanoelectrical properties of skin. Inhibition by tocopherols. Arch. Dermatol. Res..

[52] Record I.R., Dreosti I.E., Konstantinopoulos M., Buckley R.A. (1991). The influence of topical and systemic vitamin E on ultraviolet light-induced skin damage in hairless mice. Nutr. Cancer.

[53] Ritter E.F., Axelrod M., Minn K.W., Eades E., Rudner A.M., Serafin D., Klitzman B. (1997). Modulation of ultraviolet light-induced epidermal damage: beneficial effects of tocopherol. Plast. Reconstr. Surg..

[54] Roshchupkin D.I., Pistsov M.Y., Potapenko A.Y. (1979). Inhibition of ultraviolet light-induced erythema by antioxidants. Arch. Dermatol. Res..

[55] Schoonderwoerd S.A., Beijersbergen van Henegouwen G.M., Persons K.C. (1991). Effect of alpha-tocopherol and di-butyl-hydroxytoluene (BHT) on UV-A-induced photobinding of 8-methoxypsoralen to Wistar rat epidermal biomacromolecules *in vivo*. Arch. Toxicol..

[56] Yuen K.S., Halliday G.M. (1997). alpha-Tocopherol, an inhibitor of epidermal lipid peroxidation, prevents ultraviolet radiation from suppressing the skin immune system. Photochem. Photobiol..

[57] Mavon A., Raufast V., Redoules D. (2004). Skin absorption and metabolism of a new vitamin E prodrug, delta-tocopherol-glucoside: *in vitro* evaluation in human skin models. J. Control. Release.

[58] Nakayama S., Katoh E.M., Tsuzuki T., Kobayashi S. (2003). Protective effect of alpha-tocopherol-6-*O*-phosphate against ultraviolet B-induced damage in cultured mouse skin. J. Invest. Dermatol..

[59] Larsson P., Ollinger K., Rosdahl I. (2006). Ultraviolet (UV)A- and UVB-induced redox alterations and activation of nuclear factor-kappaB in human melanocytes-protective effects of alpha-tocopherol. Br. J. Dermatol..

[60] Fahlman B.M., Krol E.S. (2009). Inhibition of UVA and UVB radiation-induced lipid oxidation by quercetin. J. Agric. Food Chem..

[61] Kang J.H., Ascherio A., Grodstein F. (2005). Fruit and vegetable consumption and cognitive decline in aging women. Ann. Neurol..

[62] Selmi C., Mao T.K., Keen C.L., Schmitz H.H., Eric Gershwin M. (2006). The anti-inflammatory properties of cocoa flavanols. J. Cardiovasc. Pharmacol..

[63] Chen C.Y., Blumberg J.B. (2008). *In vitro* activity of almond skin polyphenols for scavenging free radicals and inducing quinone reductase. J. Agric. Food Chem..

[64] Li N., Jia X., Chen C.Y., Blumberg J.B., Song Y., Zhang W., Zhang X., Ma G., Chen J. (2007). Almond consumption reduces oxidative DNA damage and lipid peroxidation in male smokers. J. Nutr..

[65] Chen C.Y., Milbury P.E., Lapsley K., Blumberg J.B. (2000). Flavonoids from almond skins are bioavailable and act synergistically with vitamins C and E to enhance hamster and human LDL resistance to oxidation. J. Nutr..

[66] Suganuma M., Okabe S., Oniyama M., Tada Y., Ito H., Fujiki H. (1998). Wide distribution of [3H](-)-epigallocatechin gallate, a cancer preventive tea polyphenol, in mouse tissue. Carcinogenesis.

[67] Tardif J.C., Cote B., Lesperance J., Bourassa M., Lambert J., Doucet S., BIolodeau L., Nattel S., deGuise P. (1997). Propucol and multivitamins in the prevention of restenosis after coronary angioplasty. N. Eng. J. Med..

[68] Fortes C., Mastroeni S., Melchi F., Pilla M.A., Antonelli G., Camaioni D., Alotto M., Pasquini P. (2008). A protective effect of the Mediterranean diet for cutaneous melanoma. Int. J. Epidemiol..

[69] Purba M.B., Kouris-Blazos A., Wattanapenpaiboon N., Lukito W., Rothenberg E.M., Steen B.C., Wahlqvist M.L. (2001). Skin wrinkling: can food make a difference?. J. Am. Coll. Nutr..

[70] Neukam K., Stahl W., Tronnier H., Sies H., Heinrich U. (2007). Consumption of flavanol-rich cocoa acutely increases microcirculation in human skin. Eur. J. Nutr..

[71] Greul A.K., Grundmann J.U., Heinrich F., Pfitzner I., Bernhardt J., Ambach A., Bielsalski H.K., Gollnick H. (2002). Photoprotection of UV-irradiated human skin: an antioxidative combination of vitamins E and C, carotenoids, selenium and proanthocyanidins. Skin Pharmacol. Appl. Skin Physiol..

[72] Chiu A.E., Chan J.L., Kern D.G., Kohler S., Rehmus W.E., Kimball A.B. (2005). Double-blinded, placebo-controlled trial of green tea extracts in the clinical and histologic appearance of photoaging skin. Dermatol. Surg..

[73] Camouse M.M., Domingo D.S., Swain F.R., Conrad E.P., Matsui M.S., Maes D., Declercq L., Cooper K.D., Stevens S.R., Baron E.D. (2009). Topical application of green and white tea extracts provides protection from solar-simulated ultraviolet light in human skin. Exp. Dermatol..

[74] Yusef N., Irby C., Katiyar S.K., Elmets C.A. (2007). Photoprotective effects of green tea polyphenol. Photochem. Photoimmunol. Photomed..

[75] Mnich C.D., Hoek K.S., Virkki L.V., Farkas A., Dudli C., Laine E., Urosevic M., Dummer R. (2009). Green tea extract reduces induction of p53 and apoptosis in UVB-irradiated human skin independent of transcriptional controls. Exp. Dermatol..

[76] Aquino R., Morelli S., Tomaino A., Pellegrino M., Saija A., Grumetto L., Puglia C., Ventura D., Bonina F. (2002). Antioxidant and photoprotective activity of a crude extract of *Culcitium reflexum* H.B.K. leaves and their major flavonoids. J. Ethnopharmacol..

[77] Record I.R., Dreosti I.E. (1998). Protection by black tea and green tea against UVB and UVA + B induced skin cancer in hairless mice. Mutat. Res..

[78] Wang Z.Y., Huang M.T., Ho C.T., Chang R., Ma W., Ferraro T., Reuhl K.R., Yang C.S., Conney A.H. (1992). Inhibitory effect of green tea on the growth of established skin papillomas in mice. Cancer Res..

[79] Gensler H.L., Timmermann B.N., Valcic S., Wachter G.A., Dorr R., Dvorakova K., Alberts D.S. (1996). Prevention of photocarcinogenesis by topical administration of pure epigallocatechin gallate isolated from green tea. Nutr. Cancer.

[80] Franceschi C., Carpri M., Monti D., Giunta S., Oliverieri F., Sevini F., Panourgia M., Invidia L., Celani L., Scuriti M., Cevenini E., Castellani G.C., Salvioli S. (2007). Inflammaging and anti-inflammaging: a systemic perspective on aging and longevity emerged from studies in humans. Mech. Age. Dev..

[81] Kahraman A., Inal M.E. (2002). Protective effects of quercetin on ultraviolet A light-induced oxidative stress in the blood of rat. J. Appl. Toxicol..

[82] Casagrande R., Georgetti S.R., Verri W.A., Dorta D.J., dos Santos A.C., Fonseca M.J. (2006). Protective effect of topical formulations containing quercetin against UVB-induced oxidative stress in hairless mice. J. Photochem. Photobiol. B.

[83] Vayalil P.K., Mittal A., Hara Y., Elmets C.A., Katiyar S.K. (2004). Green tea polyphenols prevent ultraviolet light-induced oxidative damage and matrix metalloproteinases expression in mouse skin. J. Invest. Dermatol..

[84] Garbisa S., Sartor L., Biggin S., Salvato B., Benelli R., Albini A. (2001). Tumor gelatinases and invasion inhibited by the green tea flavanol epigallocatechin-3-gallate. Cancer.

[85] Morley N., Clifford T., Salter L., Campbell S., Gould D., Curnow A. (2005). The green tea polyphenol (-)-epigallocatechin gallate and green tea can protect human cellular DNA from ultraviolet and visible radiation-induced damage. Photodermatol. Photoimmunol. Photomed..

[86] Gasser P., Lati E., Peno-Mazzarino L., Bouzoud D., Allegaert L., Bernaert H. (2008). Cocoa polyphenols and their influence on parameters involved in *ex vivo* skin restructuring. Int. J. Cosmet. Sci..

[87] van Leeuwen R., Boekhoorn S., Vingerling J.R., Witteman J.C.M., Klaver C.C.W., Hofman A., de Jong P.T.V.M. (2005). Dietary intake of antioxidants and risk of age-related macular degeneration. JAMA.

[88] Krinsky N.I., Johnson E.J. (2005). Carotenoid actions and their relation to health and disease. Mol. Aspects Med..

[89] (2010). USDA USDA-NCC. Carotenoid Database for U.S. Foods-1998. USDA: Washington, DC, USA. http://www.nal.usda.gov/fnic/foodcomp/Data/car98/car98.html.

[90] Stahl W., Sies H. (1997). Antioxidant defense: Vitamins E and C and carotenoids. Diabetes.

[91] Cantrell A., McGarvey D.J., Truscott T.G., Rancan F., Bohm F. (2003). Singlet oxygen quenching by dietary carotenoids in a model membrane environment. Arch. Biochem. Biophys..

[92] Wertz K., Hunziker P.B., Seifert N., Riss G., Neeb M., Steiner G., Hunziker W., Goralczyk R. (2005). beta-Carotene interferes with ultraviolet light A-induced gene expression by multiple pathways. J. Invest. Dermatol..

[93] Peng Y.-M., Peng Y.-S., Lin Y. (1993). A nonspaonificaton method for the determination of carotenoids, retinoids, and tocopherols in solid human tissues. Cancer Epidemiol. Biomarkers Prev..

[94] Darvin M., Gersonde I., Meinke M., Sterry W., Lademann J. (2005). Non-invasive *in vivo* determination of the carotenoids beta-carotene and lycopene concentrations in the human skin using the Raman spectroscopic method. J. Phys. D: Appl. Phys..

[95] Hata T.R., Scholz T.A., Ermakov I.V., McClane R.W., Khachik F., Gellerman W., Pershing L.K. (2000). Non-invasive Raman spectroscopic detection of carotenoids in human skin. J. Invest. Dermatol..

[96] Richelle M., Sabatier M., Steiling H., Williamson G. (2006). Skin bioavailability of dietary vitamin E, carotenoids, polyphenols, bitamin C, zinc and selenium. Br. J. Nutr..

[97] Alaluf S., Heinrich U., Stahl W., Tronnier H., Wiseman S. (2002). Dietary carotenoids contribute to normal human skin color and UV photosensitivity. J. Nutr..

[98] White W.S., Kim C.I., Kalkwarf H.J., Bustos P., Roe D.A. (1988). Ultraviolet light-induced reductions in plasma carotenoid levels. Am. J. Clin. Nutr..

[99] Ribaya-Mercado J.D., Garmyn M., Gilchrest B.A., Russell R.M. (1995). Skin lycopene is destroyed preferentially over beta-carotene during ultraviolet irradiation in humans. J. Nutr..

[100] Chung H.-Y., Rasmussen H.M., Johnson E.J. (2004). Lutein bioavailability is higher from lutein-enriched eggs than from supplements and spinach in men. J. Nutr..

[101] Jacques P.F., Sulsky S.I., Sadowski J.A., Philips J.C., Rush D., Willett W.C. (1993). Comparison of micronutrient intake measured by a dietary questionnaire and biochemical indicators of micronutrient status. Am. J. Clin. Nutr..

[102] Ibiebele T.I., van der Pols J.C., Hughes M.C., Marks G.C., Williams G.M., Green A.C. (2007). Dietary pattern in association with squamous cell carcinoma of the skin: a prospective study. Am. J. Clin. Nutr..

[103] Dorgan J.F., Boakye N.A., Fears T.R., Schleicher R.L., Helsel W., Anderson C., Robinson J., Guin J.D., Lessin S., Ratnasinghe L.D., Tangrea J.A. (2004). Serum carotenoids and alpha-tocopherol and risk of nonmelanoma skin cancer. Cancer Epidemiol. Biomarkers Prev..

[104] Fung T.T., Hunter D.J., Spiegelman D., Colditz G.A., Speizer F.E., Willett W.C. (2002). Vitamins and carotenoids intake and the risk of basal cell carcinoma of the skin in women (United States). Cancer Cause. Control.

[105] Fung T.T., Spiegelman D., Egan K.M., Giovannucci E., Hunter D.J., Willett W.C. (2003). Vitamin and carotenoid intake and risk of squamous cell carcinoma of the skin. Int. J. Cancer.

[106] Rice-Evans C.A., Miller N.J., Paganga G. (1996). Structure-antioxidant activity relationships of flavonoids and phenolic acids. Free. Radic. Biol. Med..

[107] Hunter D.J., Colditz G.A., Stampfer M.J., Rosner B., Willett W.C., Speizer F.E. (1992). Diet and risk of basal cell carcinoma of the skin in a prospective cohort of women. Ann. Epidemiol..

[108] Sahl W.J., Glore S., Garrison P., Oakleaf K., Johnson S.D. (1995). Basal cell carcinoma and lifestyle characteristics. Int. J. Dermatol..

[109] van Dam R.M., Huang Z., Giovannucci E., Rimm E.B., Hunter D.J., Colditz G.A., Stampfer M.J., Willett W.C. (2000). Diet and basal cell carcinoma of the skin in a prospective cohort of men. Am. J. Clin. Nutr..

[110] Breslow R.A., Alberg A.J., Helzlsouer K.J., Bush T.L., Norkus E.P., Morris J.S., Spate V.E., Comstock G.W. (1995). Serological precursors of cancer: malignant melanoma, basal and squamous cell skin cancer, and prediagnostic levels of retinol, beta- carotene, lycopene, alpha-tocopherol, and selenium. Cancer Epidemiol. Biomarkers Prev..

[111] Davies T.W., Treasure F.P., Welch A.A., Day N.E. (2002). Diet and basal cell skin cancer: results from the EPIC-Norfolk cohort. Br. J. Dermatol..

[112] Karagas M.R., Greenberg E.R., Nierenberg D., Stukel T.A., Morris J.S., Stevens M.M., Baron J.A. (1997). Risk of squamous cell carcinoma of the skin in relation to plasma selenium, alpha-tocopherol, beta-carotene, and retinol: a nested case-control study. Cancer Epidemiol. Biomarkers Prev..

[113] Comstock G.W., Helzlsouer K.J., Bush T.L. (1991). Prediagnostic serum levels of carotenoids and vitamin E as related to subsequent cancer in Washington County, Maryland. Am. J. Clin. Nutr..

[114] Dorgan J.F., Boakye N.A., Fears T.R., Schleicher R.L., Helsel W., Anderson C., Robinson J., Guin J.D., Lessin S., Ratnasinghe L.D., Tangrea J.A. (2004). Serum carotenoids and alpha-tocopherol and risk of nonmelanoma skin cancer. Cancer Epidemiol. Biomarkers Prev..

[115] Fung T.T., Spiegelman D., Egan K.M., Giovannucci E., Hunter D.J., Willett W.C. (2003). Vitamin and carotenoid intake and risk of squamous cell carcinoma of the skin. Int. J. Cancer.

[116] Heinen M.M., Hughes M.C., Ibiebele T.I., Marks G.C., BGreen A.C., van der Pols J.C. (2007). Intake of antioxidants nutrients and th erisk of skin cancer. Eur. J. Cancer.

[117] Mathews-Roth M.M., Pathak M.A., Parrish J., Fitzpatrick T.B., Kass E.H., Toda K., Clemens W. (1972). A clinical trial of the effects of oral beta-carotene on the responses of human skin to solar radiation. J. Invest. Dermatol..

[118] Gollnick H.P.M., Hopfenmuller W., Hemmes C., Chun S.C., Schmid C., Sundermeier K., Biesalski H.K. (1996). Systemic beta-carotene plus topical UV-sunscreen are an optimal protection against harmful effects of natural UV-sunlight: results of the Berlin-Eilath study. Eur. J. Dermatol..

[119] Lee J., Jiang S., Levien N., Watson R.R. (2000). Carotenoid supplementation reduces erythema in human skin after simulated solar radiation exposure. Photochem. Photobiol..

[120] Fuller C.J., Faulkner H., Bendich A., Parker R.S., Roe D.A. (1992). Effect of β-carotene supplementation on photosupression of delayed-type hypersensitivity in normal young men. Arch. Intern. Med..

[121] Herraiz L.A., Hsieh W.C., Parker R.S., Swanson J.E., Bendich A., Roe D.A. (1998). Effect of UV and beta-carotene supplementation on delayed-type hypersensitivity response in healthy older men. J. Am. Coll. Nutr..

[122] Heinrich U., Gartner C., Wiebusch M., Eichler O., Sies H., Tronnier H., Stahl W. (2003). Supplementation with beta-carotene or a similar amount of mixed carotenoids protects humans from UV-induced erythema. J. Nutr..

[123] Kopcke W., Krutmann J. (2008). Protection from sunburn with beta-Carotene—a meta-analysis. Photochem. Photobiol..

[124] Albanes D., Virtamo J., Rautalahti M., Haukka J., Palmgren J., Gref C.G., Heinonen O.P. (1992). Serum beta-carotene before and after beta-carotene supplementation. Eur. J.Clin. Nutr..

[125] Omenn G.S., Goodman G.E., Thornquist M.D., Balmes J., Cullen M.R., Glass A., Keogh J.P., Meyskens F.L., Valais B., Williams J.H., Barnhardt S., Cherniak M.G., Brodkin C.A., Hammar S. (1996). Risk factors for lung cancer and for intervention effects in CARET, the beta-carotene and retinol efficiency trial. J. Natl. Cancer Inst..

[126] Minami Y., Kawabata K., Kubo Y., Arase S., Hirasaka K., Nikawa T., Bando N., Kawai Y., Terao J. (2009). Peroxidized cholesterol-induced matrix metalloproteinase-9 activation and its suppression by dietary beta-carotene in photoaging of hairless mouse skin. J Nutr. Biochem..

[127] Camera E., Mastrofrancesco A., Fabbri C., Daubrawa F., Picardo M., Sies H., Stahl W. (2009). Astaxanthin, canthaxanthin and beta-carotene differently affect UVA-induced oxidative damage and expression of oxidative stress-responsive enzymes. Exp. Dermatol..

[128] Mein J.R., Lian F., Wang X.-D. (2008). Biological activity of lycopene metabolites: implications for cancer prevention. Nutr. Rev..

[129] Di Mascio P., Kaiser S., Sies H. (1989). Lycopene as the most efficient biological carotenoid singlet oxygen quencher. Arch. Biochem. Biophys..

[130] Darvin M., Patzelt A., Gehse S., Schanzer S., Benderoth C., Sterry W., Lademann J. (2008). Cutaneous concentration of lycopene correlates significantly with the roughness of the skin. Eur. J. Pharm. Biopharm..

[131] Lopes L.B., VanDeWall H., Li H.T., Venugopal V., Li H.K., Naydin S., Hosmer J., Levendusky M., Zheng H., Bentley M.V.L.B., Levin R., Hass M.A. (2010). Topical delivery of lycopene using microemulsions: enhanced skin penetration and tissue antioxidant activity. J. Pharm. Sci..

[132] Stahl W., Heinrich U., Wiseman S., Eichler O., Sies H., Tronnier H. (2001). Dietary tomato paste protects against ultraviolet light-induced erythema in humans. J. Nutr..

[133] Aust O., Stahl W., Sies H., Tronnier H., Heinrich U. (2005). Supplementation with tomato-based products increases lycopene, phytofluene, and phytoene levels in human serum and protects against UV-light-induced erythema. Int. J. Vitam. Nutr. Res..

[134] Wertz K., Siler U., Goralczyk R. (2004). Lycopene: modes of action to promote prostate health. Arch. Biochem. Biophys..

[135] Basu A., Imrhan V. (2007). Tomatoes versus lycopene in oxidative stress and carcinogenesis: conclusions from clinical trials. Eur. J. Clin. Nutr..

[136] Wu W.B., Chiang H.S., Fang J.Y., Hung C.F. (2007). Inhibitory effect of lycopene on PDGF-BB-induced signalling and migration in human dermal fibroblasts: a possible target for cancer. Biochem. Soc. Trans..

[137] Handelman G.J., Nightingale Z.D., Lichtenstein A.H., Schaefer E.J., Blumberg J.B. (1999). Lutein and zeaxanthin concentrations in plasma after dietary supplementation with egg yolk. Am. J. Clin. Nutr..

[138] Mangels A.R., Holden J.M., Beecher G.R., Forman M.R., Lanza E. (1993). Carotenoid content of fruits and vegetables: an evaluation of analytic data. J. Am. Diet. Assoc..

[139] Surai P.F., MacPherson A., Speake B.K., Sparks N.H. (2000). Designer egg evaluation in a controlled trial. Eur. J. Clin. Nutr..

[140] Brown L., Rimm E.B., Seddon J.M., Giovanucci E.L., Chasen-Taber L., Speigelman D., Willett W.C., Hankinson S.E. (1999). A prospective study of carotenoid intake and risk of cataract extraction in US men. Am. J. Clin. Nutr..

[141] Chasan-Taber L., Willett W.C., Seddon J.M., Stamper M.J., Rosner B., Colditz G.A. (1999). A prospective study on vitamin supplement intake and cataract extraction among US women. Epidemiology.

[142] Seddon J.M., Ajani U.A., Sperduto R.D., Hiller R., Blair N., Burton T.C., Farber M.D., Gragoudas E.S., Haller J., Miller D.T. (1994). Dietary carotenoids, vitamins A, C, and E, and advanced age-related macular degeneration. Eye Disease Case-Control Study Group. JAMA.

[143] Mohammedshah F.D.J.S., Amann M.M., Heimbach J.M. (1999). Dietary intakes of lutein and zeaxanthin and total carotenoids among Americans age 50 and above. FASEB J..

[144] Nebeling L.C., Forman M.R., Graubard B.I., Snyder R.A. (1997). Changes in carotenoid intake in the United States: the 1987 and 1992 National Health Interview Surveys. J. Am. Diet. Assoc..

[145] Tucker K.L., Chen H., Wilson P.W.F., Schaefer E.J., Lammi-Keefe C.J. (1999). Carotenoid intakes, assessed by dietary questionnaire, are associated with plasma carotenoid concentrations in an elderly population. J. Nutr..

[146] Vandenlangenberg G.M., Brady W.E., Nebeling L.C., Block G., Forman M., Bowen P.E., Stacewicz-Sapuntzakis M., Mares-Perlman J.A. (1996). Influence of using different sources of carotenoid data in epidemiologic studies. J. Am. Diet. Assoc..

[147] Hammond B.R., Johnson E.J., Russell R.M., Krinsky N.I., Yeum K.J., Edwards R.B., Snodderly D.M. (1997). Dietary modification of human macular pigment density. Invest. Ophthalmol. Vis. Sci..

[148] Johnson E.J., Chung H.-Y., Caldarella S.M., Snodderly D.M. (2008). The influence of supplemental lutein and docosahexaenoic acid on serum, lipoproteins, and macular pigmentation. Am. J. Clin. Nutr..

[149] Johnson E.J., Hammond R.B., Yeum K.-J., Qin J., Wang X.-D., Castaneda C., Snodderly D.M., Russell R.M. (2000). Relation among serum and tissue concentrations of lutein and zeaxanthin and macular pigment density. Am. J. Clin. Nutr..

[150] Palombo P., Fabrizi G., Ruocco V., Ruocco E., Fluhr J., Roberts R., Morganti P. (2007). Beneficial long-term effects of combined oral/topical antioxidant treatment with the carotenoids lutein and zeaxanthin on human skin: a double-blind, placebo-control study. Skin Pharmacol. Physiol..

[151] Astner S., Wu A., Chen J., Philips N., Rius-Diaz F., Parrado C., Mihm M.C., Goukassian D.A., Pathak M.A., Gonzalez S. (2007). Dietary lutein/zeaxanthin partially reduces photoaging and photocarcinogenesis in chronically UVB-irradiated Skh-1 hairless mice. Skin Pharmacol. Physiol..

[152] O'Connor I., O'Brien N. (1998). Modulation of UVA light-induced oxidative stress by beta-carotene, lutein and astaxanthin in cultured fibroblasts. J. Dermatol. Sci..

[153] Chitchumroonchokchai C., Bomser J.A., Glamm J.E., Failla M.L. (2004). Xanthophylls and alpha-tocopherol decrease UVB-induced lipid peroxidation and stress signaling in human lens epithelial cells. J. Nutr..

